# Patients' recollections of experiences in the intensive care unit may affect their quality of life

**DOI:** 10.1186/cc3026

**Published:** 2005-01-31

**Authors:** Cristina Granja, Alice Lopes, Sara Moreira, Claudia Dias, Altamiro Costa-Pereira, António Carneiro, for the JMIP Study Group

**Affiliations:** 1 Intensivist, Consultant in Anesthesiology, Medical Intensive Care Unit, Hospital Pedro Hispano, Matosinhos, Portugal; 2 Consultant in Psychiatry, Department of Psychiatry, Hospital Geral de Santo Antonio, Oporto, Portugal; 3 Research Assistant, Department of Biostatistics and Medical Informatics, Faculty of Medicine, University of Oporto, Oporto, Portugal; 4 Professor and Head of Department, Department of Biostatistics and Medical Informatics, Faculty of Medicine University of Oporto, Oporto, Portugal; 5 Consultant in Internal Medicine, Head of Department of Intensive Care, Intensive Care Unit, Hospital Geral de Santo António, Oporto, Portugal

**Keywords:** critical illness, follow-up, health-related quality of life, intensive care, neuropsychological sequelae, outcome

## Abstract

**Introduction:**

We wished to obtain the experiences felt by patients during their ICU stay using an original questionnaire and to correlate the memories of those experiences with health-related quality of life (HR-QOL).

**Methods:**

We conducted a prospective study in 10 Portuguese intensive care units (ICUs). Six months after ICU discharge, an original questionnaire on experiences of patients during their ICU stay, the recollection questionnaire, was delivered. HR-QOL was evaluated simultaneously, with the EQ-5D questionnaire. Between 1 September 2002 and 31 March 2003 1433 adult patients were admitted. ICU and hospital mortalities were 21% and 28%, respectively. Six months after ICU discharge, 464 patients completed the recollection questionnaire.

**Results:**

Thirty-eight percent of the patients stated they did not remember any moment of their ICU stay. The ICU environment was described as friendly and calm by 93% of the patients. Sleep was described as being good and enough by 73%. The experiences reported as being more stressful were tracheal tube aspiration (81%), nose tube (75%), family worries (71%) and pain (64%). Of respondents, 51% experienced dreams and nightmares during their ICU stay; of these, 14% stated that those dreams and nightmares disturb their present daily life and they exhibit a worse HR-QOL. Forty-one percent of patients reported current sleep disturbances, 38% difficulties in concentrating in current daily activities and 36% difficulties in remembering recent events. More than half of the patients reported more fatigue than before the ICU stay. Multiple and linear regression analysis showed that older age, longer ICU stay, higher Simplified Acute Physiology Score II, non-scheduled surgery and multiple trauma diagnostic categories, present sleep disturbances, daily disturbances by dreams and nightmares, difficulties in concentrating and difficulties in remembering recent events were independent predictors of worse HR-QOL. Multicollinearity analysis showed that, with the exception of the correlation between admission diagnostic categories and length of ICU stay (0.47), all other correlations between the independent variables and coefficient estimates included in the regression models were weak (below 0.30).

**Conclusion:**

This study suggests that neuropsychological consequences of critical illness, in particular the recollection of ICU experiences, may influence subsequent HR-QOL.

## Introduction

Patients admitted to an intensive care unit (ICU) generally present an unexpected life-threatening condition, with the exception of those admitted after scheduled surgery. These patients will remain in their critical condition for various lengths of time and will need several types of life support, such as ventilation, cardiovascular or renal support. They will also receive various types of sedatives and analgesics to ensure compliance with ventilation and to induce some comfort. As the event that takes these critical patients to the ICU was unexpected, most patients will not be aware of their condition until late in their ICU stay and some of them only after their discharge to the ward. However, during their ICU stay they continue to have an emotional life, in a mixture of dreams, delusions and emotional experiences related to real events.

Although various degrees of anxiety or depression that might delay and impair their recovery have been described in critical illness survivors [1-4], little is known about this and other neuropsychological sequelae of critical illness; cognitive impairment and memory disturbances are those more frequently described [1-5]. Post-traumatic stress disorder (PTSD) [6] and PTSD-related symptoms (PTSS) [2] have also been described as possible events occurring after critical illness.

Although functional sequelae seem to depend more on previous health state and on the existence of co-morbidities and on the aggressiveness suffered during the critical illness period, neuropsychological sequelae depend not only on the aggressiveness of the acute event but also on the ability of patients to deal with the memories they retain from that period [1-3]. These memories may be of two kinds: factual memories and delusional memories, which include nightmares, hallucinations, paranoid delusions and dreams [2]. Recall of delusional memories but not of factual memories has been associated with the development of acute PTSS [2].

Several studies have sought to identify factors that can function as stressors during an ICU stay, with the aim of preventing or decreasing them [7-10].

This study has two aims: to recollect the experiences felt by patients during their ICU stay, by using an original questionnaire, and to correlate the memories of those experiences with health-related quality of life (HR-QOL).

## Methods

This study is part of a multicentre study on the quality of life after intensive care, involving 10 Portuguese ICUs; these are listed in Additional file 1 and have been named the Jornadas de Medicina Intensiva da Primavera (JMIP) Study Group.

### Patients

The study addressed all adult patients (aged 18 years or more) admitted to the 10 ICUs. Background variables included patient's gender, age, main activity and previous health state. On the basis of individual clinical registries and on direct questioning from patients for whom a follow-up consultation was continuing, the previous health state was evaluated according to three categories: healthy, chronic non-disabling diseases (that is, able to perform work or normal daily activities) and chronic disabling diseases (that is, unable to work or to undertake normal daily activities). Each participating physician in each ICU classified all patients into one of these three categories. ICU variables included the severity of disease at admission as evaluated by Simplified Acute Physiology Score II (SAPS II), the length of stay and the admission diagnostic category (medical, scheduled surgery, non-scheduled surgery or multiple trauma).

### Methods

The first author developed an original questionnaire to recollect experiences lived by survivors of critical illness, which was called the recollection questionnaire (see Additional file 2) and was based on previous personal experience with an ICU follow-up clinic [11-14] and previous studies on this subject [2,7,8]. The questions were extensively applied over several years by the first author and changes were made over time to achieve the best possible understanding from the patient about each proposed question. The questionnaire was therefore developed after a succession of small pilot and qualitative studies.

The recollection questionnaire comprises 14 questions relating to memories retained by the patients, the environment in the ICU, the relationship with health care professionals, dreams, nightmares, sleep disturbances, difficulties in concentrating and in remembering recent events, fatigue and being able to return to their previous level of activity. Direct questions on memories were made either on real experiences of patients in the ICU or on dreams and nightmares experienced by them. There was no formal division between factual memories and delusional memories. Hallucinations or paranoid delusions were not specifically looked for. One of the questions (number 11) comprises 25 items related to the recollection of experiences lived in the ICU, such as tracheal suctioning, needle punctures, pain, sleep, and dependence on the ventilator. These items can be classified in one of five categories: 0 ('I don't remember'), 1 ('It was not hard'), 2 ('It was indifferent'), 3 ('It was hard but necessary'), 4 ('It was very hard'), and 5 ('It was awful').

HR-QOL was measured with a generic questionnaire (EQ-5D) [15,16] and a specific critical care questionnaire [17]. For the purpose of this study, only data of the generic questionnaire will be reported. EQ-5D is a generic instrument designed to measure health outcome that was developed at the European level [15,16]. The EuroQol Group originally developed the Portuguese version of EQ-5D in 1998 (EuroQol Group Newsletter, January 2000). EQ-5D was applied as reported previously [11].

At 6 months after discharge from ICU, all recollection questionnaires were sent by mail. For practical reasons all patients completed their questionnaires at home. In five ICUs questionnaires were returned by mail and in the other five they were returned directly by hand when patients came to the follow-up consultation.

Informed consent was obtained from all patients at the time of the follow-up consultation, where applicable. Also, because questionnaires were sent by mail, a letter containing detailed information on the aims of the study accompanied them. Thus, because consent was implicit in answering the questionnaire, the need for additional informed consent was waived. A hospital Ethics Committee approved this observational study.

Descriptive analyses of background variables (gender, age, main activity and previous health state), ICU variables (SAPS II, length of ICU stay and admission diagnostic category) and questionnaire variables were presented. Categorical variables were described as absolute frequencies (*n*) and relative frequencies (%); median and centiles were used for continuous variables. The Pearson test, linear-by-linear test and Mann–Whitney test were used for comparisons.

Multiple logistic regression was performed with the five dimensions of the EQ-5D questionnaire as dependent variables (categorised as not having problems or having problems) and background, ICU and recollection questionnaire variables as independent variables. The stepwise Forward method was used with an entry criterion of *P *< 0.05 and a removal criterion of *P *< 0.1. To analyse possible multicollinearity between the variables studied, Spearman correlation coefficients between the variables and regression coefficient estimates correlation matrices were analysed.

Differences were considered statistically significant at *P *< 0.05. SPSS^® ^12.0 was used for statistical analysis.

## Results

**Figure 1:**
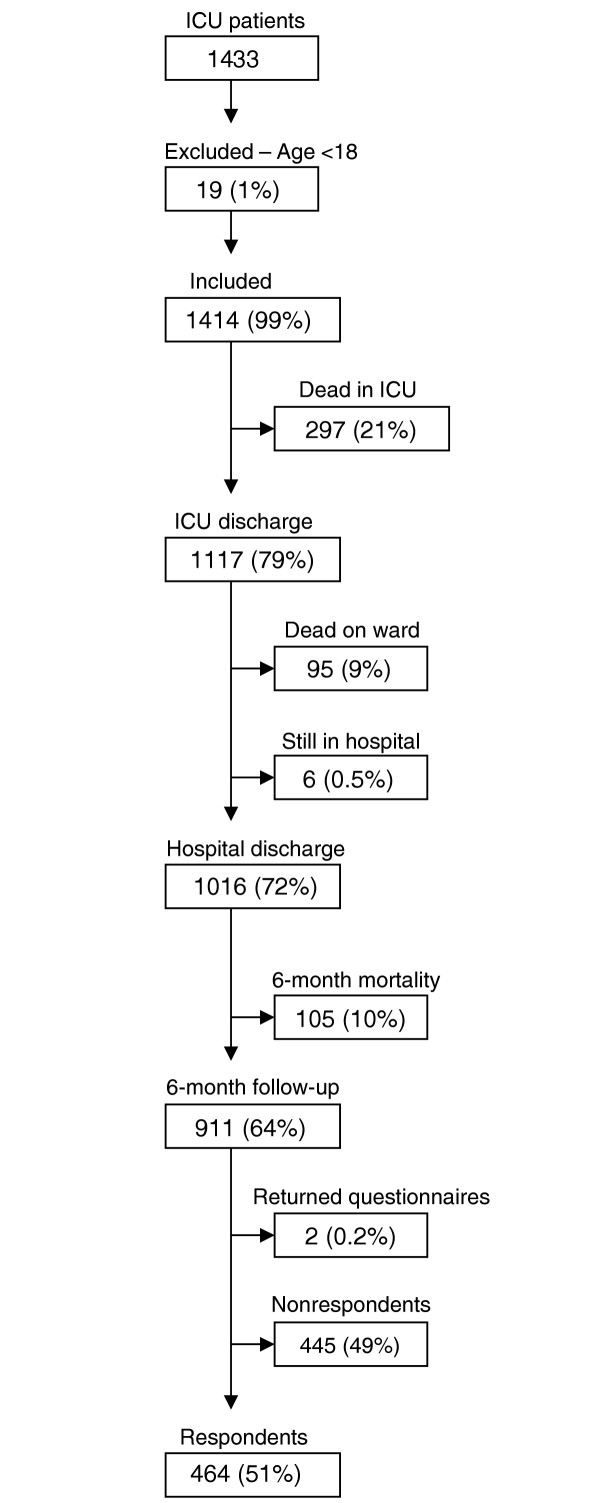
Patients included in and excluded from the study. Survival and recollection questionnaire response rates.

Between 1 September 2002 and 31 March 2003 there were 1433 admissions. Nineteen patients were excluded because they were less than 18 years old. Two hundred and ninety-seven (21%) died in the ICU and a further 95 patients died in the ward (28% in-hospital mortality rate). At 6 months, six patients were still in the hospital. One hundred and five patients died after hospital discharge but before the evaluation at 6 months, at which point there were 911 survivors, 445 (49%) of them being non-respondents. Four hundred and sixty-four patients completed the recollection questionnaire (Fig. 1).

There were no differences between respondents and non-respondents in background and ICU variables, except for admission diagnostic category, for which non-scheduled surgery and multiple trauma survivors answered significantly less (Table 1).

### Background variables

Of the 464 respondents included in the study, 61% were male, the median age was 58 years, 49% were retired, 39% were previously healthy, 44% had previous chronic non-disabling disease and 17% had previous chronic disabling disease (Table 1).

### ICU variables

The median SAPS II on admission was 31, the median length of ICU stay was 4 days and admission diagnostic categories in ICU included medical reasons in 46% of the patients, scheduled surgery in 32%, non-scheduled surgery in 13% and multiple trauma in 9% (Table 1).

With the exception of gender and length of ICU stay, which exhibited non-significant differences, there was significant variability between the 10 ICUs: the minimum median age was 44 years and the maximum was 68 (*P *= 0.016), those reporting their main activity as employed varied between 12% and 50% (*P *= 0.011), previous health state varied between 6% and 66% previously healthy (*P *= 0.001), median SAPS II exhibited a minimum of 26 and a maximum of 39 (*P *= 0.004), and diagnostic categories varied for medical admissions between 25% and 71% (*P *< 0.001; Table 2).

### Recollection questionnaire variables

There was also significant variability between the 10 ICUs in the answers to the recollection questionnaire, as follows: of the 464 respondents, 23% stated that they had amnesia about hospital admission (range 6–42%), and 45% stated that they had amnesia about ICU admission (range 21–68%). Moreover, when asked about remembering some moment during their ICU stay (question 3), 38% (range 20–55%) stated that they had amnesia about the whole ICU stay. For purposes of data analysis these 38% of patients will be assumed to be those who had amnesia about the ICU stay (Table 3).

Of those who remembered (*n *= 236; question 3), the ICU environment was described as friendly and calm by 93% (range 63–100%) of the patients. Confidence in ICU physicians and ICU nurses was described as being excellent by 94% (range 82–100%) and good by 96% (range 81–100%) of the patients. Sleep in the ICU was described as excessive by 11% (range 0–20%) of the patients, enough and restoring by 62% (range 31–82%) of the patients, and insufficient by 27% (range 0–56%) (Table 3).

When asked about their own perception of their quality of life, 40% (range 10–82%) considered that it had improved, 31% (range 0–84%) that it remained the same, 20% (range 0–31%) that it worsened, 1% (range 0–6%) would have preferred to die and 8% (range 0–19%) did not know how to answer (Table 3). Patients who considered that they had improved or remained the same as before the ICU stay exhibited significantly fewer problems in all dimensions of the EQ-5D, and a significantly higher EQ-VAS and EQ Index (data not shown).

Eighty percent of patients had never before been admitted to an ICU. Being previously admitted to an ICU was significantly associated with being retired, previous chronic disease, medical diagnostic categories, and a report of problems in the anxiety/depression dimension (data not shown).

Concerning the 25 items in question 11 (see Additional file 2), where patients were asked to classify experiences according to the degree of stress provoked, to simplify the analysis we combined those items classified as 1 and 2 as being not stressful and those classified as 3, 4 and 5 as being stressful. Table 4 shows the recollection of experiences reported as being more stressful (that is, difficult to endure): tracheal tube aspiration (81%), nose tube (75%), family worries (71%), pain (64%), immobilisation in bed (64%), fear of dying or uncertainty about the future (64%), daily needle punctures (61%), difficulties in communication (59%), machine (ventilator) dependence (58%), general discomfort (58%), bladder tube (56%) and noisy and non-sleeping nights (54%).

Comparing background, ICU and EQ-5D variables between those who remembered some moment in the ICU (62%) and those with amnesia (38%), we found that those remembering some moment in the ICU exhibited significantly fewer problems in the mobility, self-care and usual activities dimensions, had significantly higher EQ-VAS and EQ Index and stated themselves to be better in a significantly higher percentage, although those who exhibited amnesia were also significantly more severely ill and stayed significantly longer in the ICU (data not shown).

Fifty-four percent of patients who were not retired were unable to return to their previous level of activity, and 51% of those who were retired were also not able to return to their previous level of activity (Table 3).

From all respondents, 41% experienced dreams and 30% experienced nightmares during their ICU stay (Table 3). Combining the patients with these experiences, we found no significant differences between background and ICU variables in those who did not experience dreams and nightmares, but those who experienced dreams and nightmares reported significantly more problems in the pain/discomfort and anxiety/depression dimensions (data not shown). Fourteen percent (*n *= 23) of these respondents stated that those dreams and nightmares disturb their current daily life (that is, at 6 months after ICU discharge). Although not exhibiting statistically significant differences in the background and ICU variables, they reported significantly more moderate to extreme problems in the pain/discomfort dimension (91% versus 55%) and in the anxiety/depression dimension (77% versus 51%). They also exhibited a statistically significantly lower EQ-VAS and EQ Index (Table 5).

Forty-one percent of the patients reported current sleep disturbances (Table 3). Sleep disturbances were significantly associated with female gender, older age, being retired and a worse HR-QOL in all the dimensions of the EQ-5D, including a significantly worse EQ-VAS and EQ Index (data not shown).

Thirty-eight percent of patients reported difficulties with concentrating in present daily activities (Table 3), and these were significantly associated with being retired and a worse HR-QOL in all dimensions of the EQ-5D, including EQ-VAS and EQ Index (data not shown).

Thirty-six percent of patients reported difficulties in remembering recent events (Table 3), and these were significantly associated with being retired, severity of disease at ICU admission and a worse HR-QOL in all dimensions of the EQ-5D including EQ-VAS and EQ Index (data not shown).

Fifty-seven percent of patients reported more fatigue at 6 months than before the ICU stay (Table 3), and these exhibited a significantly worse HR-QOL in all dimensions of the EQ-5D, including a significantly worse EQ-VAS and EQ Index, although there were no significant differences in the background and ICU variables (data not shown). Fatigue was significantly associated with the ability to return to their previous level of activity. These patients exhibited a significantly small rate of return to their previous level of activity, both those who were employed and even those who were retired (data not shown).

With multiple logistic regression analysis we found that older age, longer ICU stay, higher SAPS II, non-scheduled surgery and trauma admission diagnostic categories were, as expected, independent predictors of the report of problems in the dimensions of the EQ-5D (Table 6). It was also found that current sleep disturbances, current dreams and nightmares that disturb daily life, difficulties in concentrating and difficulties in remembering recent events were all independent predictors of the report of problems in the dimensions of the EQ-5D (Table 6).

Multiple linear regression analysis of EQ-VAS and EQ Index showed that older age, higher SAPS II, having dreams and nightmares that disturb daily life, difficulties in concentrating and difficulties in remembering recent events were significantly associated with a lower EQ-VAS and EQ Index (data not shown).

Multicollinearity analysis showed that, with the exception of the correlation between admission diagnostic categories and length of ICU stay (0.47), all other correlations between the independent variables and coefficient estimates included in the five regression models were weak (below 0.30; data not shown).

## Discussion

In this study, nearly a half of the patients did not remember the moment of their admission to the ICU, although this percentage fell to 38% when they were asked whether they remembered some moment in their ICU stay. This agrees with previous studies in which 21–30% of patients exhibited amnesia about their ICU stay [8,9]. We found that amnesia was associated with a worse HR-QOL; however, that association was no longer significant in multiple regression analysis. A previous study by Jones and colleagues [2] has suggested that memories of factual events may protect against subsequent PTSS, whereas delusional memories were associated with more anxiety/depression. Results from the present study might suggest the same protective effect of remembering the ICU stay.

Nearly half of the survivors reported dreams and nightmares during their ICU stay and a smaller percentage of these patients (14%) reported still being disturbed by them at 6 months after ICU discharge. These patients exhibited a significantly worse HR-QOL, particularly in the pain/discomfort and anxiety/depression dimensions. In addition, the report of current disturbance to their daily life by those dreams and nightmares might suggest PTSS.

Results from multiple logistic and linear regression analyses showed that current sleep disturbances, difficulties in concentrating and difficulties in remembering recent events at 6 months after ICU discharge were all significantly associated with a worse HR-QOL, indicating a common platform of neuropsychological sequelae in survivors of critical illness involving cognitive problems, memory disturbances and anxiety/depression disturbances; this finding has been described in previous studies [2,3,5,6,18,19]. Multicollinearity analysis suggested that these items might, in fact, be independent predictors of a worse HR-QOL. In a previous study in survivors of cardiac arrest from our ICU, at the follow-up evaluation we found that about half of the survivors exhibited cognitive dysfunction, including memory deficits and problems in executive functions [20], which drew our attention to the need for neurocognitive evaluation of survivors of intensive care.

Tracheal tube aspiration, nose tube, family worries and pain were the ICU experiences described as being more stressful. Neuropsychological consequences in ICU survivors have been described as being related either to environmental factors (characteristic of the ICU, which can lead to an overwhelming of sensory stimuli) or factors related to memory problems (namely delusional memories and amnesia) [2,3,5,18,19]. These findings should suggest a need not only to review our concepts of optimal analgesia and sedation but also to evolve strategies to reinforce and help maintain factual memories, such as dialogue with the patients, explanation of all procedures, maintenance of the day/night cycle, minimisation of sensory stimuli and minimisation of noise and lights. Although for some patients the noise of alarms and seeing staff around them may be reassuring, trying to make the ICU a quiet place, at least during the night, seemed to us a good strategy.

About 65% of those patients who reported more fatigue at 6 months than before their ICU stay did not return to their previous level of activity/employment. This reinforces the fact that these consequences can have an independent effect on the ability of patients to return to work, and thus have a socio-economic impact [1].

Patients' own perception of quality of life significantly correlates with all the domains of EQ-5D, a finding similar to that of Eddleston and colleagues with the Short-Form Health Survey (SF-36) [3], which indicates the usefulness of HR-QOL generic instruments on this population.

The use of specific measurement tools for cognitive disturbances, for post-traumatic stress-related symptoms and for anxiety/depression (not done in this study) would overcome some limitations regarding the identification of these specific sequelae. Kapfhammer and colleagues [21] recently published a study in which they used specific tools to look for psychiatric morbidity and its influence on the HR-QOL of survivors of acute respiratory distress syndrome. The authors established a significant association between the diagnosis of PTSD at follow-up and more unfavorable values in the most important psychosocial dimensions of SF-36.

This study presents some other limitations, as follows.

1. There was a relatively high non-response rate (49%); however, we did not find any statistically significant differences with regard to background and ICU variables between respondents and non-respondents, including previous health state and severity of illness at ICU admission. Thus, other factors might partly contribute to the non-response rate, such as a significant proportion of functional illiteracy. Furthermore, for most of the 10 ICUs, follow-up consultations were something completely new in the evaluation of patients, which might also partly contribute to the relatively high non-response rate.

2. The recollection questionnaire was not formally assessed for its face or content validity. Although the questionnaire was developed after a succession of small pilot and qualitative studies, as stated above, we acknowledge the potential limitation caused by the lack of more formal reproducibility and validity studies.

3. As the multicentre study followed a continuing study in the first author's ICU, we did not apply a standardised tool that was meanwhile developed by Jones and coworkers (Intensive Care Unit Memory tool) [22] as our study progressed. This standardised tool was subsequently applied by Capuzzo and coworkers and was recently published [23].

4. We were unable to collect information regarding either restraint protocols or sedation protocols in the different ICUs.

5. Because specific tools for the evaluation of anxiety, depression or PTSS were not used, we could not establish further findings with regard to not only their own characterisation but also their role on the neuropsychological consequences of intensive care and on their relationships with HR-QOL at 6 months after ICU discharge.

Four main findings may be drawn from this study, as follows.

1. As a multicentre study, it enabled us to understand a core of problems common to all our ICUs, which should draw our attention to specific neuropsychological sequelae from illness requiring critical care.

2. The study contributed to identifying which experiences were reported as responsible for more stress during their ICU stay, a crucial issue in trying to identify and reduce stress factors. Tracheal tube aspiration, nose tube, pain and immobilisation in bed were stressors notably common to the experiences previously described in other studies [7-10,23,24]. In addition, family worries were the third factor identified as responsible for stress in our patients. This can be explained by the traditionally strong family ties in the Portuguese culture. Pain came in fourth place in the ranking of stressors. This finding, together with the need to preserve factual memories [2,18], should encourage revision of analgesia/sedation strategies in accordance with more recent guidelines [25].

3. Amnesia about ICU stay, sleep disturbances at 6 months after ICU stay, and memory and cognitive disturbances were associated with a worse HR-QOL, indicating not only specific neuropsychological sequelae but also their influence on subsequent HR-QOL.

4. About 15% of patients reported dreams and nightmares during their ICU stay, and these patients also exhibited a worse HR-QOL at 6 months after ICU discharge, as measured by EQ-5D. Although we did not look for hallucinations or paranoid delusions, this finding is in accordance with findings of Jones and colleagues [2], linking delusional memories with the development of PTSS and a worse HR-QOL. The association between current memory disturbances, cognitive disturbances, sleep disturbances and subsequent quality of life may be one of the key messages from this study.

## Conclusion

This study suggests that neuropsychological consequences of critical illness might affect subsequent HR-QOL, which should direct our attention to these consequences and encourage further research.

## Key messages

• 	The study contributed to identifying which experiences were reported as responsible for more stress during their ICU stay: tracheal tube aspiration, nose tube, family worries, pain, immobilization in bed, and fear of dying/uncertainty in the future, were the most frequent stress factors reported by patients.

• 	The association between current memory disturbances, cognitive disturbances, sleep disturbances and subsequent quality of life may be one of the key messages from this study.

• 	This study suggests that neuropsychological consequences of critical illness might affect subsequent HR-QOL, which should direct our attention to these consequences and encourage further research.

## Abbreviations

HR-QOL = health-related quality of life; ICU = intensive care unit; LOS = length of ICU stay; PTSD = post-traumatic stress disorder; PTSS = PTSD-related symptoms; SAPS = Simplified Acute Physiology Score.

## Competing interests

The author(s) declare that they have no competing interests.

## Authors' contributions

CG created and designed the study and was responsible for the final manuscript. AL and SM advised for the search of neuropsychological consequences in critical patients and contributed to the final interpretation of these consequences on the final manuscript. CD undertook the statistical analysis. ACP conducted the statistical analysis and wrote the final manuscript. AC contributed to the design and the coordination of the study. All authors read and approved the final manuscript.

## Supplementary Material

Additional File 1 List of the ICUs participating in the JMIP Study Group. Click here for file

Additional File 2 Table containing the recollection questionnaire. Click here for file

## Figures and Tables

**Table 1:** Comparison of background and intensive care unit variables between respondents and non-respondents

Variable	Total (*n *= 909)	Respondents (*n *= 464)	Non-respondents (*n *= 445)	*P*
Background data				
Sex, *n *(%)				
Male	535 (59)	281 (39)	254 (42)	0.286^a^
Female	374 (41)	183 (61)	254 (57)	
Median age, years (P25–P75)	59 (42–70)	58 (43–69)	60 (41–72)	0.212^b^
Main activity, *n *(%)				
Employed	128 (29)	128 (29)	-	
Retired	216 (49)	216 (49)	-	
Housework/student/seeking work	58 (12)	58 (12)	-	
Other	44 (10)	44 (10)	-	
Previous health state, *n *(%)				
Healthy	371 (41)	182 (39)	189 (42)	0.228^a^
Chronic non-disabling disease	403 (44)	203 (44)	200 (45)	
Chronic disabling disease	135 (15)	78 (17)	57 (13)	
ICU variables				
Median SAPS II at admission (P25–P75)	32 (22–42)	31 (22–41)	33 (22–43)	0.209^b^
Median days in ICU (P25–P75)	4 (2–10)	4 (2–10)	5 (2–10)	0.297^b^
Admission category, *n *(%)				
Medical	417 (46)	214 (46)	203 (46)	0.011^a^
Scheduled surgery	247 (27)	144 (32)	103 (23)	
Non-scheduled surgery	138 (15)	62 (13)	76 (17)	
Multiple trauma	106 (12)	44 (9)	62 (14)	

^a ^Pearson χ^2^. ^b ^Mann–Whitney test. ICU, intensive care unit; SAPS, Simplified Acute Physiology Score. P25 and P75 are the 25th and 75th centiles.

**Table 2:** Background and intensive care unit variables from the 10 intensive care units

Variable	Total (*n *= 464)	ICU 1 (*n *= 39)	ICU 2 (*n *= 74)	ICU 3 (*n *= 38)	ICU 4 (*n *= 66)	ICU 5 (*n *= 44)	ICU 6 (*n *= 32)	ICU 7 (*n *= 59)	ICU 8 (*n *= 16)	ICU 9 (*n *= 54)	ICU 10 (*n *= 42)	*P*
Background data												
Sex, *n *(%)												
Male	281 (61)	25 (64)	46 (62)	22 (58)	37 (56)	30 (62)	19 (59)	32 (54)	10 (62)	34 (63)	26 (62)	0.995^a^
Female	183 (39)	14 (36)	28 (38)	16 (42)	29 (44)	14 (32)	13 (41)	27 (46)	6 (38)	20 (37)	16 (38)	
Median age, years (P25–P75)	58 (43–69)	44 (32–55)	55 (44–69)	65 (56–71)	53 (39–56)	59 (41–68)	55 (28–67)	62 (48–73)	68 (54–77)	62 (47–73)	57 (43–69)	0.016^b^
Main activity, *n *(%)												
Employed	128 (29)	14 (38)	19 (26)	6 (17)	28 (45)	11 (26)	8 (27)	12 (21)	2 (12)	7 (13)	21 (50)	0.011^a^
Retired	216 (48)	12 (32)	36 (49)	19 (54)	24 (39)	24 (56)	12 (40)	30 (53)	12 (75)	33 (65)	14 (33)	
Housework/student/seeking work	58 (13)	6 (16)	9 (12)	7 (20)	5 (8)	3 (7)	7 (23)	8 (14)	2 (12)	5 (10)	6 (14)	
Other	44 (10)	5 (14)	9 (12)	3 (9)	5 (8)	5 (11)	3 (10)	7 (12)	0 (0)	6 (12)	1 (2)	
Previous health state, *n *(%)												
Healthy	183 (39)	19 (49)	16 (22)	10 (26)	28 (42)	19 (43)	21 (66)	31 (52)	1 (6)	15 (28)	23 (55)	<0.001^a^
Chronic non-disabling disease	203 (44)	13 (33)	51 (69)	23 (61)	19 (29)	12 (27)	11 (34)	20 (34)	11 (69)	32 (59)	11 (26)	
Chronic disabling disease	78 (17)	7 (18)	7 (9)	5 (13)	19 (29)	13 (30)	0 (0)	8 (14)	4 (25)	7 (13)	8 (19)	
ICU variables												
Median SAPS II at admission (P25–P75)	31 (22–41)	31 (17–40)	30 (17–39)	31 (24–46)	26 (19–35)	31 (18–42)	38 (27–44)	30 (22–37)	28 (21–47)	31(23–42)	39 (29–52)	0.004^b^
Median days in ICU (P25–P75)	4 (2–10)	7 (2–11)	1 (1–3)	5 (1–9)	5 (2–10)	4 (2–8)	10 (6–17)	4 (1–11)	2 (1–6)	3 (1–9)	7 (3–12)	0.434^b^
Admission category, *n *(%)												
Medical	214 (46)	16 (41)	24 (32)	27 (71)	28 (42)	19 (43)	21 (66)	26 (44)	4 (25)	31 (57)	18 (43)	<0.001^a^
Scheduled surgery	144 (32)	3 (8)	42 (57)	10 (26)	23 (35)	18 (41)	2 (6)	17 (29)	10 (63)	10 (19)	9 (21)	
Non-scheduled surgery	62 (13)	9 (23)	6 (8)	1 (3)	13 (20)	7 (16)	3 (9)	12 (20)	1 (6)	5 (9)	5 (12)	
Multiple trauma	44 (9)	11 (28)	2 (3)	0 (0)	2 (3)	0 (0)	6 (19)	4 (7)	1 (6)	8 (15)	10 (24)	

^a ^Pearson χ^2^. ^b ^Mann–Whitney test. ICU, intensive care unit; SAPS, Simplified Acute Physiology Score. P25 and P75 are the 25th and 75th centiles.

**Table 3:** Results from the recollection questionnaire

Question	Number (% of responses)
Do you remember your admission to the hospital?	
Yes	319 (77)
Do you remember your admission to the intensive care unit (ICU)?	
Yes	230 (55)
Regarding what you saw and felt during your ICU stay:	
I prefer not to remember	52 (14)
I don't remember anything	143 (38)
I don't mind remembering	137 (36)
I what to remember everything	34 (9)
None of them	13 (3)
How would you describe the environment in the ICU?	
Friendly and calm	189 (93)
Chaotic and terrifying	4 (2)
Hostile and tense	11 (5)
Your confidence in doctors was:	
Excellent	122 (53)
Good	93 (41)
Sufficient	12 (5)
Bad	2 (1)
Your confidence in nurses was:	
Excellent	118 (52)
Good	100 (44)
Sufficient	8 (3)
Bad	2 (1)
How do you classify your sleep during ICU stay?	
Excessive	24 (11)
Enough and restoring	140 (62)
Insufficient	62 (27)
At 6 months after ICU stay your quality of life:	
Has improved	162 (40)
Is the same	125 (31)
Is worse	81 (20)
I would prefer to have died	6 (1)
I don't know how to answer	34 (8)
Had you been previously admitted to an ICU?	
Once for the same reason	33 (8)
Twice or more for the same reason	12 (3)
Once for a different reason	38 (9)
Never been admitted to an ICU before	320 (80)
If you are not retired, have you returned to your previous activity?	
No	101 (54)
*If not*: Because of ICU stay	42(47)
If you are retired, have you returned to your previous activity?	
No	91 (51)
*If not*: Because of ICU stay	33 (42)
Have you had many dreams during the ICU stay?	
Yes	139 (41)
Did you have many nightmares during the ICU stay?	
Yes	98 (30)
Currently, do you remember those dreams and nightmares?	
Yes	83 (49)
Currently, do you think that those dreams and nightmares disturb your daily life?	
Yes	23 (14)
Currently, do you have sleep disturbances?	
Yes	153 (41)
Currently, do you have difficulties in concentrating?	
Yes	139 (38)
Currently, do you have difficulties in remembering recent events?	
Yes	136 (36)
Currently, do you feel more fatigue than before the ICU stay?	
Yes	199 (57)

**Table 4:** Recollection of stressful experiences in the intensive care unit, according to the classification defined in the recollection questionnaire

		Remember^a ^		
			
Experience	*n*	With stress, *n *(%)	Without stress, *n *(%)	Amnesia^b ^*n *(%)
Daily needle punctures	362	114 (61)	72 (39)	176 (49)
Tracheal tube aspiration	326	113 (81)	26 (19)	187 (57)
Nose tube	343	127 (75)	42 (25)	174 (51)
Bladder tube	330	90 (56)	72 (44)	166 (50)
Noise from conversation	351	31 (17)	146 (83)	174 (50)
Noise from engines and ventilators	360	63 (32)	132 (68)	165 (46)
Pain	360	121 (64)	69 (36)	170 (47)
Bedridden	347	129 (64)	73 (36)	145 (42)
Music in the intensive care unit	339	14 (12)	100 (88)	225 (66)
Comments from doctors and nurses	351	20 (13)	128 (87)	203 (58)
Noisy and bad sleeping nights	349	83 (54)	71 (46)	195 (56)
Ventilator dependence	343	93 (58)	68 (42)	182 (53)
Dependence on doctors and nurses	347	71 (39)	110 (61)	166 (48)
Lack of privacy in hygiene	347	79 (43)	103 (57)	165 (48)
Communication difficulties	349	111 (59)	78 (41)	160 (46)
Brightness from artificial lights	348	56 (33)	116 (67)	176 (51)
Fear of being disconnected from the ventilator	321	41 (41)	58 (59)	222 (69)
General discomfort	340	98 (58)	71 (42)	171 (50)
Fear of dying, uncertain of the future	353	110 (64)	62 (36)	181 (51)
Medical round near the patient's bed	346	13 (7)	163 (93)	170 (49)
Fear of medical procedures	342	35 (20)	139 (80)	168 (49)
Losing time orientation	348	56 (37)	94 (63)	198 (57)
Family worries	352	129 (71)	53 (29)	170 (48)
Economic worries	339	59 (38)	95 (62)	185 (55)

^a ^Refers to patients who remembered their stay in the intensive care unit.

^b ^Refers to all respondents.

**Table 5:** Comparison of background, intensive care unit and EQ-5D variables between those who stated that dreams and nightmares from the intensive care unit currently disturbed their daily life and those who did not

Variable		Disturbance by dreams and nightmares		
		
	Total (*n *= 169)	No (*n *= 146)	Yes (*n *= 23)	*P*
Background data				
Sex, *n *(%)				
Male	95 (56)	82 (56)	13 (56)	0.974^a^
Female	754 (44)	64 (44)	10 (44)	
Median age (P25–P75)	52 (41–67)	51 (40–67)	57 (45–66)	0.434^2^
Main activity, *n *(%)				
Employed	57 (35)	50 (35)	7 (32)	0.654^a^
Retired	70 (42)	60 (42)	10 (45)	
Housework/student/seeking work	20 (12)	15 (10)	5 (23)	
Other	17 (11)	18 (13)	0 (0)	
Previous health state, *n *(%)				
Healthy	78 (46)	68 (47)	10 (43)	0.798^a^
Chronic non-disabling disease	58 (34)	48 (33)	10 (43)	
Chronic disabling disease	33 (20)	30 (20)	3 (14)	
ICU variables				
Median SAPS II at admission (P25–P75)	31 (22–40)	31 (22–40)	26 (22–35)	0.208^b^
Median ICU days (P25–P75)	5 (2–11)	6 (2–11)	3 (1–7)	0.071^b^
Admission category, *n *(%)				
Medical	82 (49)	73 (50)	9 (39)	0.450^a^
Scheduled surgery	46 (27)	38 (26)	8 (35)	
Non-scheduled surgery	24 (14)	22 (15)	2 (9)	
Multiple trauma	17 (10)	13 (9)	4 (17)	
EQ-5D variables				
Mobility, *n *(%)				
N: I have no problems in walking about	90 (54)	83 (58)	7 (32)	0.042^c^
M: I have some problems in walking about	74 (45)	59 (41)	15 (68)	
E: I am confined to bed	2 (1)	2 (1)	0 (0)	
Self-care, *n *(%)				
N: I have no problems with self-care	111 (67)	101 (70)	10 (45)	0.084^c^
M: I have some problems washing or dressing myself	41 (25)	31 (22)	10 (45)	
E: I am unable to wash or dress myself	14 (8)	12 (8)	2 (10)	
Usual activities, *n *(%)				
N: I have no problems with performing my usual activities	61 (37)	56 (39)	5 (24)	0.183^c^
M: I have some problems with performing my usual activities	75 (46)	64 (44)	11 (52)	
E: I am unable to perform my usual activities	29 (18)	24 (17)	5 (24)	
Pain/discomfort, *n *(%)				
N: I have no pain or discomfort	67 (40)	65 (45)	2 (9)	<0.001^c^
M: I have moderate pain or discomfort	81 (49)	68 (47)	13 (59)	
E: I have extreme pain or discomfort	18 (11)	11 (8)	7 (32)	
Anxiety/depression, *n *(%)				
N: I am not anxious or depressed	74 (45)	69 (49)	5 (23)	0.009^c^
M: I am moderately anxious or depressed	63 (38)	53 (37)	10 (45)	
E: I am extremely anxious or depressed	27 (17)	20 (14)	7 (32)	
Perceived current health state				
Health state today compared with 12 months ago, *n *(%)				
Better	78 (47)	70 (49)	8 (36)	0.119^c^
The same	51 (31)	45 (31)	6 (27)	
Worse	37 (22)	29 (20)	8 (36)	
Median EQ-VAS on a 100% scale (P25–P75)	65 (50–80)	70 (50–81)	50 (40–60)	0.001^b^
Median EQ Index (P25–P75)	67 (49–91)	72 (50–91)	45 (35–67)	0.002^b^

^a ^Pearson χ^2^. ^b ^Mann–Whitney test. ^c ^Linear-by-linear association. ICU, intensive care unit; SAPS, Simplified Acute Physiology Score. P25 and P75 are the 25th and 75th centiles.

**Table 6:** Results from five regression models

Variable	OR	95% CI
Mobility		
Age	1.03	1.01–1.05
LOS	1.07	1.02–1.11
SAPS II	1.01	0.99–1.03
Difficulties in concentrating		
No	1.00	
Yes	1.79	0.965–3.33
Difficulties in remembering recent events		
No	1.00	
Yes	2.14	1.14–4.01
Self-care		
Age	1.02	1.00–1.04
LOS	1.04	1.00–1.08
SAPS II	1.02	1.00–1.04
Dreams and nightmares disturb your daily life		
No	1.00	
Yes	3.32	1.09–10.08
Difficulties in concentrating		
No	1.00	
Yes	3.55	1.99–6.35
Usual activities		
Age	1.04	1.02–1.05
LOS	1.08	1.03–1.13
Difficulties in concentrating		
No	1.00	
Yes	6.27	3.29–11.91
Pain/discomfort		
Age	1.03	1.01–1.05
Admission category		
Scheduled surgery	1.00	
Non-scheduled surgery	3.90	1.51–10.08
Medical	1.72	0.93–3.16
Multiple trauma	5.57	1.83–16.91
Dreams and nightmares disturb your daily life		
No	1.00	
Yes	11.39	1.39–93.33
Sleep disturbances		
No	1.00	
Yes	2.54	1.46–4.42
Anxiety/depression		
Dreams and nightmares disturb your daily life	1.00	
No	4.91	1.00–23.95
Yes		
Sleep disturbances		
No	1.00	
Yes	2.49	1.38–4.51
Difficulties in concentrating		
No	1.00	
Yes	2.53	1.32–4.83
Difficulties remembering recent events		
No	1.00	
Yes	2.58	1.37–4.86

Dependent variables were the five dimensions of the EQ-5D questionnaire. Independent variables were all background and intensive care unit variables, and questions 12, 13 and 14 from the recollection questionnaire.

CI, confidence interval; LOS, length of ICU stay; OR, odds ratio; SAPS, Simplified Acute Physiology Score.
