# Supplementary material for: Patients' recollections of experiences in the intensive care unit may affect their quality of life
Source: Crit Care. 2005 Jan 31;9(2):R96–R109. doi: 10.1186/cc3026 (PMC1175917; doi:10.1186/cc3026)
Supplement: Additional File 1 — List of the ICUs participating in the JMIP Study Group. [file cc3026-S1.pdf]

Additional file 1

The JMIP (Jornadas de Medicina Intensiva da Primavera) Study Group.

Participants:

Ana Isabel Paixão (Centro Hospitalar de Gaia - V.N.Gaia); António Carneiro (Hospital Geral de Santo António - Porto); Cristina Granja (Hospital Pedro Hispano - Matosinhos); Eduardo Silva (Hospital do Desterro - Lisboa); Helena Estrada (Hospital Santo António dos Capuchos - Lisboa); José Vaz (Hospital José Joaquim Fernandes - Beja); Lurdes Gonçalves (Hospital de Vila Real - Vila Real); Paula Coutinho (Centro Hospitalar de Coimbra - Coimbra); Paulo Martins (Hospital Universitário de Coimbra - Coimbra); Piedade Amaro (Hospital de S. Sebastião - Sta. Maria da Feira); Cláudia Dias e Altamiro Costa Pereira (Serviço Biostatística e Informática Médica da Faculdade de Medicina da Universidade do Porto - Porto).
