# Supplementary material for: Patients' recollections of experiences in the intensive care unit may affect their quality of life
Source: Crit Care. 2005 Jan 31;9(2):R96–R109. doi: 10.1186/cc3026 (PMC1175917; doi:10.1186/cc3026)
Supplement: Additional File 2 — Table containing the recollection questionnaire. [file cc3026-S2.pdf]

Additional file 2

Recollection questionnaire – Patients' recollection of ICU experiences

Taking into account what you remember from your ICU stay, please tick the appropriate box on each question. Please not that one question should be signaled with only one tick.

1. Do you remember your admission on hospital? Yes □ No □

2. Do you remember your admission on the Intensive Care Unit (ICU)? Yes □ No □

3. Relative to what you have seen and felt during your ICU stay:

1.I prefer not to remember □ 2.I don't remember anything □ 3.I don't mind to remember □

4. I want to remember everything □ 5.None of them □

4. How do you describe the environment in the ICU?

1. Friendly and calm □ 2. Caotic and terrifying □ 3. Hostil and tense □ 4. Idon´t remember □

5. **Your confidence in doctors was**: Excellent □ Good □ Sufficient □ Bad □

6. **Your confidence in nurses was**: Excellent □ Good □ Sufficient □ Bad □

7. How do you classify your sleep during ICU stay:

1. Excessive □ 2. Sufficient and restoring □ 3. Insufficient □

8. At six months after ICU stay your quality of life

1. Has improved □ 2. Is the same □ 3. Is worst □ 4. I preferred to have died □ 5. I don't know how to answer □

9. Have you been previously admitted in an ICU?

1. Once for the same reason □
2. Twice or more for the same reason □
3. Once for a different reason □
4. Never been admitted in an ICU □

10.a) If you are not retired have you returned to your previous activity? Yes □ No □

If not: 1. because of ICU stay □

2. For another reason not related with ICU stay□

b) If you are retired, have you returned to your previous activity Yes □ No □

If not: 1. because of ICU stay □

2. For another reason not related with ICU stay□

11. Bearing in mind how difficult to endure was to you (i.e. how stressful) each of the following procedures/events during your ICU stay, please answer to the following questions according to the classification:

0 - I don’t remember	3 - It was hard, but it had to be
1 - It was not hard	4 - It was very hard
2 - It was indifferent	5 - It was awful

a.	Daily needle punctures	0	1	2	3	4	5
b.	Tracheal tube aspiration	0	1	2	3	4	5
c.	Nose tube	0	1	2	3	4	5
d.	Bladder tube	0	1	2	3	4	5
e.	Noise from conversation	0	1	2	3	4	5
f.	Noise from engines and ventilators	0	1	2	3	4	5
g.	Pain	0	1	2	3	4	5
h.	Bedridden	0	1	2	3	4	5
i.	Music in the ICU	0	1	2	3	4	5
j.	Comments from doctors and nurses	0	1	2	3	4	5
k.	Noisy and bad sleeping nights	0	1	2	3	4	5
l.	Ventilator dependence	0	1	2	3	4	5
m.	Doctors and nurses dependence	0	1	2	3	4	5
n.	Lack of privacy in hygiene	0	1	2	3	4	5
o.	Communication difficulties	0	1	2	3	4	5
p.	Brightness from artificial lights	0	1	2	3	4	5
q.	Fear of dying, uncertainty in the future	0	1	2	3	4	5
r.	General discomfort	0	1	2	3	4	5
s.	Fear of being disconnected from the ventilator	0	1	2	3	4	5
t.	Medical rounds near the patient’s bed	0	1	2	3	4	5
u.	Fear of medical procedures	0	1	2	3	4	5
v.	Loosing time orientation	0	1	2	3	4	5
w.	Family worries	0	1	2	3	4	5
x.	Economic worries	0	1	2	3	4	5

12. a) **Have you had many dreams during ICU stay?** Yes ☐ No ☐

b) Have you had many nightmares during ICU stay? Yes ☐ No ☐

13. a) **Currently, do you remember those dreams and nightmares?** Yes ☐ No ☐

b) Currently, do you think that those dreams and nightmares disturb your daily life? Yes ☐ No ☐

c) Currently, do you have sleep disturbances? Yes ☐ No ☐

d) Currently, do you have difficulties in concentrating? Yes ☐ No ☐

e) Currently, do you have difficulties in remembering recent events? Yes ☐ No ☐

14. **Currently, do you feel more fatigue than previously to ICU stay?** Yes ☐ No ☐
